# Electrical, Thermal, and Structural Characterization of Plant-Based 3D Printed Gel Polymer Electrolytes for Future Electrochemical Applications

**DOI:** 10.3390/polym15244713

**Published:** 2023-12-15

**Authors:** Muhammad Afiq Hazizi Mahamood, Muhammad Faishal Norjeli, Ahmad Adnan Abu Bakar, Shahino Mah Abdullah, Nizam Tamchek, Ikhwan Syafiq Mohd Noor, Ala H. Sabeeh, Ahmad Fudy Alforidi, Ibrahim H. Khawaji, Mohd Ifwat Mohd Ghazali

**Affiliations:** 1 SMART RG, Faculty of Science and Technology, Universiti Sains Islam Malaysia, Bandar Baru Nilai, Nilai 71800, Negeri Sembilan, Malaysia; afiqhazizi97@raudah.usim.edu.my (M.A.H.M.);; 2 Department of Physics, Faculty of Science, Universiti Putra Malaysia, Serdang 43400, Selangor Darul Ehsan, Malaysia; 3 Physics Division, Centre of Foundation Studies for Agricultural Science, Universiti Putra Malaysia, Serdang 43400, Selangor Darul Ehsan, Malaysia; 4 Department of Electrical Engineering, Taibah University, P.O. Box 344, Al-Madina Al Munawara 42353, Saudi Arabia; asabeeh@taibahu.edu.sa (A.H.S.); aforidi@taibahu.edu.sa (A.F.A.);

**Keywords:** 3D printing, stereolithography (SLA), gel polymer electrolyte (GPE), lithium perchlorate (LiClO_4_), plant-based polymer

## Abstract

In this work, a plant-based resin gel polymer electrolyte (GPE) was prepared by stereolithography (SLA) 3D printing. Lithium perchlorate (LiClO_4_) with a concentration between 0 wt.% and 25 wt.% was added into the plant-based resin to observe its influence on electrical and structural characteristics. Fourier transform infrared spectroscopy (FTIR) analysis showed shifts in the carbonyl, ester, and amine groups, proving that complexation between the polymer and LiClO4 had occurred. GPEs with a 20 wt.% LiClO_4_ (S20) showed the highest room temperature conductivity of 3.05 × 10^−3^ S cm^−1^ due to the highest number of free ions as determined from FTIR deconvolution. The mobility of free ions in S20 electrolytes was also the highest due to greater micropore formation, as observed via field emission scanning electron microscopy (FESEM) images. Transference number measurements suggest that ionic mobility plays a pivotal role in influencing the conductivity of S20 electrolytes. Based on this work, it can be concluded that the plant-based resin GPE with LiClO_4_ is suitable for future electrochemical applications.

## 1. Introduction

Over the last few years, the demand for energy has expanded significantly due to the rapid growth in the global population and changes in consumer preferences. Energy storage systems are needed to harvest energy from various sources and convert it into a form of energy for use in many applications, such as electrical appliances, utilities, and transportation. Energy storage systems can be used to balance the supply and demand for energy. There are numerous forms of energy storage systems, such as thermal energy storage, thermochemical energy storage, and electrochemical energy storage [1]. Electrochemical energy storage consists of supercapacitors and batteries [2]. Nowadays, batteries are advanced applications with high voltage, high energy density, large charge numbers and discharge cycles [3], and portability. Due to their outstanding performance, batteries are widely used in electronic devices and biomedical applications [4].

Generally, a battery consists of three main components which are an anode, cathode, and electrolyte. An electrolyte is one of the important parts of batteries; it acts as a separator and a medium for ionic transportation between the electrodes. When liquid electrolytes (LEs) are used in batteries, the risk of solvent evaporation, electrochemical corrosion, and leakage is always present [5]. To address these issues, various polymer electrolyte systems have been studied and applied in numerous electrochemical devices. There are three categories of polymer electrolytes: composite polymer electrolytes, solid polymer electrolytes (SPEs), and gel polymer electrolytes (GPEs) [6]. Solid polymer electrolytes are being considered as an alternative to conventional liquid electrolytes due to their thermal stability, flexibility, chemical and physical stability, and lack of leakage. Despite the advantages of SPEs over LEs, they show poor ionic conductivity [7]. Therefore, GPEs are preferred over SPEs for practical applications in batteries because they combine high ionic conductivity with adequate mechanical properties, which are based on LEs and SPEs [8]. GPE has emerged as one of the most favored electrolytes for the fabrication of modern energy storage devices, possessing increased safety and flexibility [9]. Furthermore, GPE typically has a room temperature ionic conductivity of up to 10^−3^ S cm^−1^ [10], which is comparable to that of commercial liquid electrolytes.

In essence, polymer materials are needed to prepare GPEs. Polymethyl methacrylate (PMMA) [11], polyvinylidene fluoride (PVDF) [12], polyacrylonitrile (PAN) [13], polyethylene oxide (PEO) [14], polyvinyl butyl (PVB) [15], and polyurethane acrylate (PUA) [16] are the polymer hosts commonly used in GPE fabrication. Apart from these polymers, plant-based polymers have received much interest over the past few decades due to their environmental friendliness. Plant-based polymers are typically similar to polymers developed naturally by living things [17]. The properties of this type of polymer include the following: employment of natural resources as the primary component, non-toxicity, biodegradability, and sustainability [18]. Furthermore, there are various types of plant-based polymers, such as cellulose, starch, chitosan, latex, and vegetable oils. Vegetable oils are mainly made from palm oil, castor oil, and soybean oil, and they are used for resins, coatings, and adhesives [19]. The process for developing plant-based polymer resins usually begins with the extraction of vegetable oil before the polymers undergo chemical modification such as hydroxylation, epoxidation, acrylation, isocyanation, and transesterification [20]. Due to the benefits that plant-based polymers possess, they act as polymer hosts to produce polymer electrolytes. As in previous studies, a plant-based polymer electrolyte incorporated with lithium perchlorate (LiClO_4_) was successfully prepared to study the effect of salt content on electrolyte properties and achieved an optimal room temperature conductivity of 1.29 × 10^−4^ S cm^−1^ [21].

An abundance of research towards the advancement of the material and preparation method of GPE has been conducted to boost its performance, especially in terms of ionic conductivity. One of the latest advancements is the integration of additive manufacturing, also known as 3D printing, into GPE fabrication. This technique fabricates components in a layer-by-layer manner from computer-aided design (CAD) files which allow for the production of intricate geometrical and customizable structures [22]. Furthermore, 3D printing is classified into various techniques including material jetting, binder jetting, powder bed fusion, sheet lamination, material extrusion, directed energy deposition, and vat photopolymerization [23]. All these types have their own materials, benefits, and disadvantages which must be further emphasized so that they can be fully used in the proposed application.

Stereolithography (SLA) is one type of vat photopolymerization method that is frequently used in additive manufacturing processes due to its good compromise between speed and high printing resolution [24]. SLA is a layer-by-layer photopolymerization technique in which a photosensitive resin is polymerized with ultraviolet (UV) light [25]. Photopolymer resin consists of photo initiators, monomers, and oligomers. During photopolymerization, photo initiators are initiated by the laser to generate free radicals, causing them to generate crosslinking reactions between functionalized oligomers and monomers to form solids [26]. SLA and other types of additive manufacturing have three major elements: 3D printers, materials, and computer-aided design (CAD). If some changes are made to these elements, they could alter the properties or functionality of the 3D printed outcomes. The choice of materials for SLA is currently limited since the method requires a photopolymer [27]. Plant-based photopolymer is one of the materials that has attracted much interest because it is environmentally friendly, abundant in nature, and it also has non-toxic properties, biodegradability features, and ecological benignity [28].

The deployment of a plant-based photopolymer as a polymer matrix for GPE fabrication through a 3D printing SLA method would bring a great added value to the GPE. However, it has not been achieved in any studies. In this work, 3D printed plant-based GPE was prepared by the SLA method with epoxidized resin-based soybean oil. The effects of various lithium perchlorate (LiClO_4_) concentrations on the electrical, structural, and thermal properties of 3D printed plant-based GPE were investigated.

## 2. Materials and Methods

### 2.1. Materials

**Figure 1:**
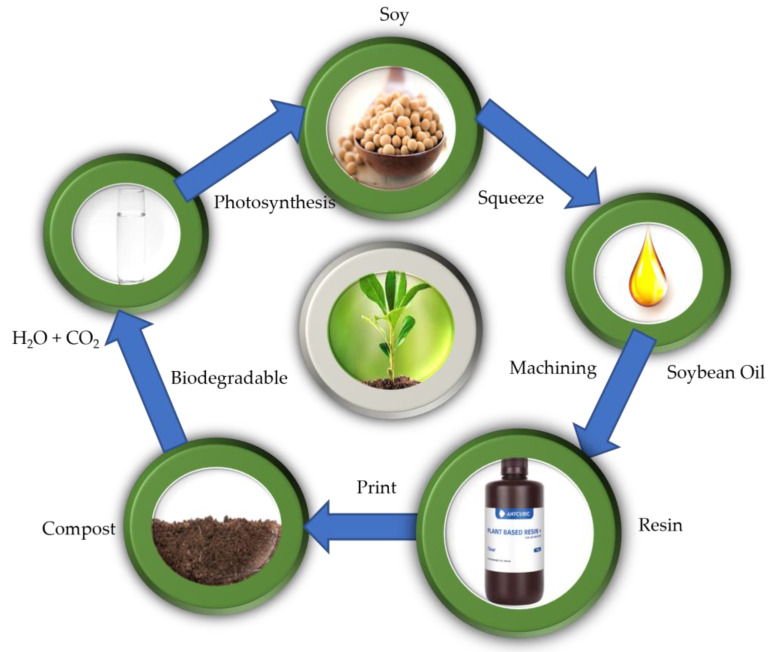
Anycubic plant-based resin is biodegradable and truly eco-friendly.

Photosensitive Anycubic plant-based resin with a weight of 0.5 kg is a biodegradable UV clear resin made from epoxidized soybean oil, which serves as a polymer host as shown in Figure 1 below. It was purchased from Anycubic Technology Co. Limited, Hongkong, China, and its compositions are listed in Table 1. Lithium perchlorate (LiClO_4_) and dimethyl formamide (DMF) were obtained from Sigma Aldrich. All reagents and chemicals were used exactly as given. 

Table 2 lists several comparisons between regular resin and plant-based resin. Regular resin has a stronger smell than plant-based resin. The ingredients in regular resin are originally made from industrial chemicals, while plant-based resin is made from soybean oil. In addition, plant-based resin has a wider compatibility in the wavelength range as it is sensitive to UV light of 355 nm to 405 nm. In terms of environmental friendliness, regular resin degrades more slowly than plant-based resin when buried in the soil.

### 2.2. Preparation of 3D Printing Plant-Based Gel Polymer Electrolyte by Stereolithography

**Figure 2:**
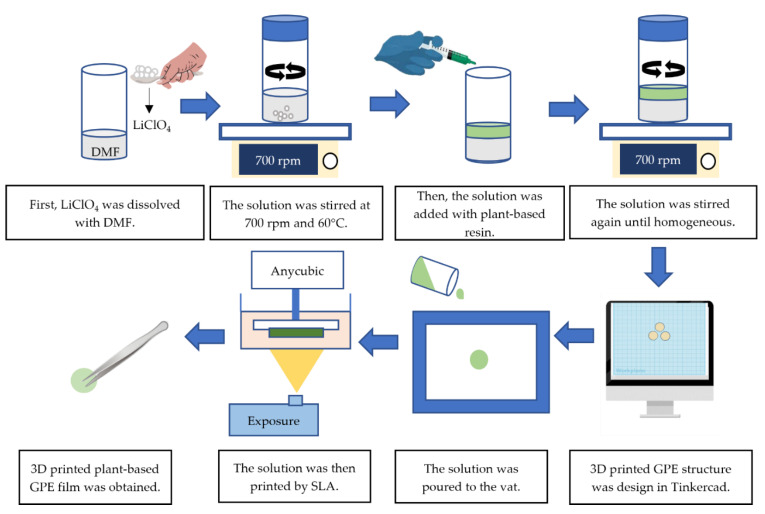
Fabrication process of 3D printed plant-based GPE film.

**Figure 3:**
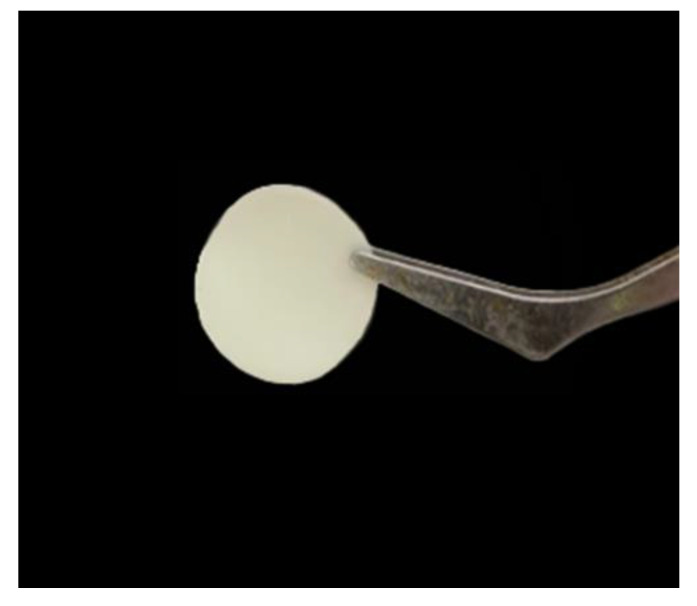
3D printed plant-based GPE.

To prepare the 3D printed plant-based gel polymer electrolyte (GPE), different concentrations of LiClO_4_ as listed in Table 3 were stirred with DMF acting as a solvent in variant vials for 24 h at a temperature of 60 °C and at a rotation speed of 700 rpm by a magnetic stirrer. Next, 2 g of photosensitive Anycubic plant-based resin was added to the electrolyte solution and stirred continuously until a homogeneous mixture was formed. The mixed solution was then poured onto the Anycubic photon S printer vat/tank and cured by ultraviolet (UV) light via a photopolymerization process. Before printing, Tinkercad, an online 3D modelling application, was used to create 3D printing files. The modeling designs were converted to a standard triangle language (.stl) file format and transferred into the Photon Workshop slicer program (Anycubic, Shenzhen, China), where they were sliced according to the selected settings as shown in Table 4 [30]. The stereolithography (SLA) method takes 10 min to prepare the 3D printed plant-based GPE thin film; the film was rinsed with isopropanol (IPA). Then, the samples were used for analysis. Figure 2 shows the fabrication process of the 3D printed plant-based GPE film whilst the 3D printed plant-based GPE film was successfully fabricated as shown in Figure 3. 

### 2.3. Characterizations

Electrochemical impedance spectroscopy (EIS) was used to determine the ionic conductivity of the 3D printed plant-based GPEs. The sample was sandwiched between a pair of stainless steel block electrodes. The stainless steel electrodes were then connected to a Hioki 3532-50 LCR Hi-tester (Nagona, Japan). A frequency range between 50 Hz and 1 MHz [31] and a 0.01 V amplitude were applied across the sample, and the impedance was recorded. A graph of negative imaginary (−*Z*″) against real (*Z*′) impedance or known as a Nyquist plot was then plotted. The bulk resistance (*R_b_*) was obtained from the Nyquist plot, and the ionic conductivity (*σ*) of each sample was determined using Equation (1).

(1) $$\;\sigma =\frac{t}{R_{b}A}.$$

In Equation (1), *A* is known as the surface area of the plant-based GPE films in contact with the electrode (2.65 cm^2^), and *t* is the thickness of the GPEs (0.05 cm). From the impedance data, real (*ε*′) and imaginary (*ε*″) permittivity values as a function of frequency for a sample with various LiClO_4_ concentrations were deduced. The *ε*′ and *ε*″ can be deduced using Equations (2) and (3).

(2) $$\;\varepsilon^{’}=\frac{Z^{\prime \prime }}{\omega C_{o}\left(Z^{’2}+Z^{\prime \prime 2}\right)},$$

(3) $$\;\varepsilon^{\prime \prime }=\frac{Z^{’}}{\omega C_{o}\left(Z^{’2}+Z^{\prime \prime 2}\right)}.$$

Here, $C_{o}=\frac{\varepsilon_{o}A}{t}$, $\varepsilon_{o}$= free space permittivity, *A* = the surface area of plant-based GPE films contacted with the electrode, *t* = thickness of plant-based GPE films and *ω* = 2π*f* (*f* = frequency in hertz). The real (*M*’) and imaginary (*M*″) electrical modulus can be obtained using Equations (4) and (5):

(4) $$\;M^{’}=\frac{\varepsilon^{’}}{\left(\varepsilon^{’2}+\varepsilon^{\prime \prime 2}\right)},$$

(5) $$\;M^{\prime \prime }=\frac{\varepsilon^{\prime \prime }}{\left(\varepsilon^{’2}+\varepsilon^{\prime \prime 2}\right)}.$$

To identify the effect of salt on the 3D printed plant-based GPE complex system, FTIR measurements were performed. The Thermo Fisher Scientific model Nicolet iS50 (Waltham, MA, USA) was used to measure the IR spectrum of each sample’s wavenumber from 4000 to 500 at room temperature with a 4 cm^−1^ resolution. From the spectrum, the percentage of free ions and ion pairs can be estimated by deconvoluting the band between 610 cm^−1^ and 650 cm^−1^, referring to the characteristics of ClO_4_^−^ free ions in this region. Equations (6) and (7) were used to calculate the percentage of free ions (*f_ions_*) and ion pairs (*P_ions_*), respectively.

(6) $$\;f_{ions}\left(\%\right)=\frac{A_{f}}{A_{f}+A_{p}}\times 100,$$

(7) $$\;P_{ions}\left(\%\right)=\frac{A_{p}}{A_{f}+A_{p}}\times 100.$$

In Equations (6) and (7), *A_f_* refers to the free ion area and *A_p_* refers to the ion pair area.

From the impedance data, number density (*n*), mobility (*μ*) and diffusion coefficient (*D*) of charge carriers were deduced. Equations (8)–(10) were used to estimate *n*, *μ* and *D* for all prepared electrolytes.

(8) $$\;n=\frac{\left(\frac{m_{salt}}{M_{w}}\times N_{A}\right)}{V_{T}}\times \%f_{ions}\times 2,$$

(9) $$\;\mu =\frac{\sigma }{ne},$$

(10) $$\;D=\frac{\mu k_{b}T}{e}.$$

In Equations (8)–(10), $m_{salt}$ = mass of LiClO_4_, $M_{w}$ = molecular weight, $N_{A}$ = the Avogadro constant, $V_{T}\;\;$= total volume, *e =* charges in the electron, $k_{b}$ = the Boltzmann constant, $\%f_{ions}$ = the area percentage of free ions and *T* = temperature in kelvin.

Transference number measurement (TNM) in plant-based GPE was estimated to determine whether the conductivity of the sample was more anionic or cationic. The sample was sandwiched between a pair of stainless steel electrodes, and a constant dc voltage of 1.5 V was applied across the sample. The dc current flow in the sample was determined as a function of time. Equations (11) and (12) were then used to determine the transference numbers from the polarization current against the time plot [32]:

(11) $$\;t_{ion}=\frac{I_{i}-I_{f}}{I_{i}},$$

(12) $$\;t_{ele}=\frac{I_{f}}{I_{i}}.$$

Here, *I_f_* is the final remaining current and $I_{i}$ is the starting current.

Thermogravimetric analysis (TGA) was performed using TGA550 (New Castle, DE, USA) to determine the thermal stability of the prepared sample. The 3D printed plant-based GPEs were heated from 50 °C to 600 °C at a 20 mL min^−1^ flow rate and 10 °C min^−1^ under nitrogen atmosphere. Differential scanning calorimetry (DSC) was carried out to identify the glass phase transition of 3D printed plant-based GPEs. DSC250 (New Castle, DE, USA) was used to perform the measurements. A sample of 4 mg was placed in an alumina pan and heated from −20 to 200 °C with a 10 °C min^−1^ scan rate under a 20 mL min^−1^ flow rate of nitrogen gas.

Field emission scanning electron microscopy (FESEM) model JEOL JSM-7800F (Singapore) was used to analyze the morphology of 3D printed plant-based GPEs. FESEM with a 2500 magnification and a 0.50 kV electron beam were applied to investigate the microspore surfaces of 3D printed GPE.

Considering the issue of environmental friendliness, a biodegradation test was carried out. The pure plant-based polymer weighed at 0.1711 g was buried in peat moss soil. The biodegradability of the pure plant-based polymer was tested using 100 g of peat moss soil and the soil characteristics were obtained before the polymer was planted in the soil. Upon planting the pure plant-based polymer in the soil, the film was rinsed with isopropanol (IPA) and dried at room temperature. The weight loss percentage of the sample was determined every 5 days, and it was left to degrade for about 40 days. The following equation was used to determine the weight loss percentage:

(13) $$\mathrm{Weight}\;\mathrm{loss}\;(\%)=\frac{W_{i}-W_{f}}{W_{i}}\times 100.$$

In Equation (13), $W_{i}$ indicates the plant-based polymer’s early weight and $W_{f}$ defines the weight just after the planting test [33].

## 3. Results and Discussion

### 3.1. Electrochemical Impedance Spectroscopy (EIS)

**Figure 4:**
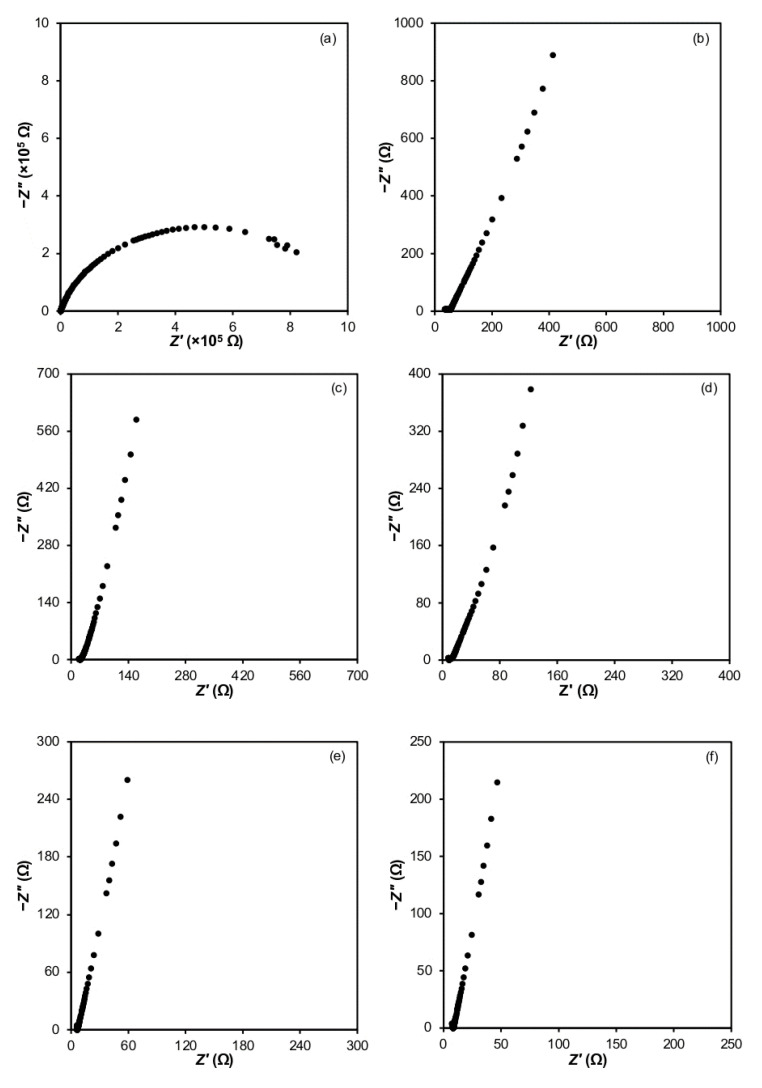
Nyquist plots for (**a**) S0, (**b**) S5, (**c**) S10, (**d**) S15, (**e**) S20 and (**f**) S25 electrolytes.

EIS analysis was performed to study the electrical properties of 3D printed plant-based GPEs. Figure 4 shows the Nyquist plot for plant-based GPEs consisting of 0 wt.% to 25 wt.% LiClO_4_. It can be seen that all samples have the shape of a depressed semicircle at a high frequency range and a tilted spike at a low frequency range. Plant-based GPE’s bulk resistance ($R_{b}$) was determined from the interception of the depressed semicircle with the tilted spike. The $R_{b}$ value obtained was substituted into Equation (1) to calculate the electrolyte ionic conductivity (*σ*). Table 5 lists the values of $R_{b}$ and *σ* for 3D printed plant-based GPEs. It is noted that the bulk resistance decreases as the amount of lithium salt increases.

**Figure 5:**
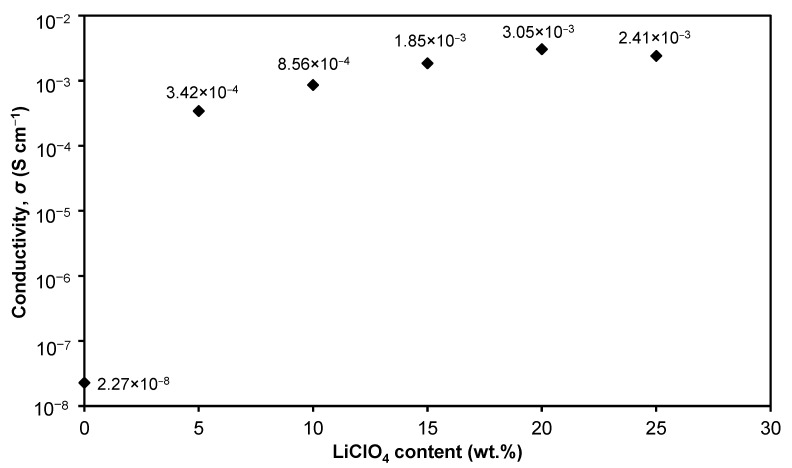
Conductivity of plant-based 3D printed GPE with various LiClO_4_ concentrations.

In this work, LiClO_4_ was chosen as the dopant salt. LiClO_4_ is commonly referred to as a great ionic salt due to its outstanding dissociation capabilities (lattice energy of 380.99 kJ mol^−1^), negligible resistive properties, and fewest electronegative properties [34]. Table 5 and Figure 5 depict the correlation between the $R_{b}$ and *σ* of 3D printed plant-based GPEs with different LiClO_4_ salt concentrations. As shown in Figure 5, it can be observed that the conductivity of a free salt electrolyte (S0 electrolyte) is 2.27 × 10^−8^ S cm^−1^. The addition of a 5 wt.% LiClO_4_ increases the conductivity of the electrolyte to four orders of magnitude which is 3.42 × 10^−4^ S cm^−1^ (S5 electrolyte). The conductivity of the plant-based 3D printed GPE system is seen to increase gradually until it reaches a maximum of 3.05 × 10^−4^ S cm^−1^ for a sample consisting of 20 wt.% salt (S20 electrolyte). The increase in conductivity after the addition of LiClO_4_ salt to plant-based 3D printed electrolyte can be attributed to the increase in charge carriers within the system [35]. When LiClO_4_ salt was added to a concentration of more than 20 wt.% (S25 electrolyte), the conductivity of ions decreased. This circumstance can be explained by the decrease in the number of free ions due to the ion association and ion pair production [36]. Ion pairs are neutral, thus not contributing to conductivity resulting in the decrease in ionic conductivity after a 20 wt.% LiClO_4_ as seen in Figure 5.

**Figure 6:**
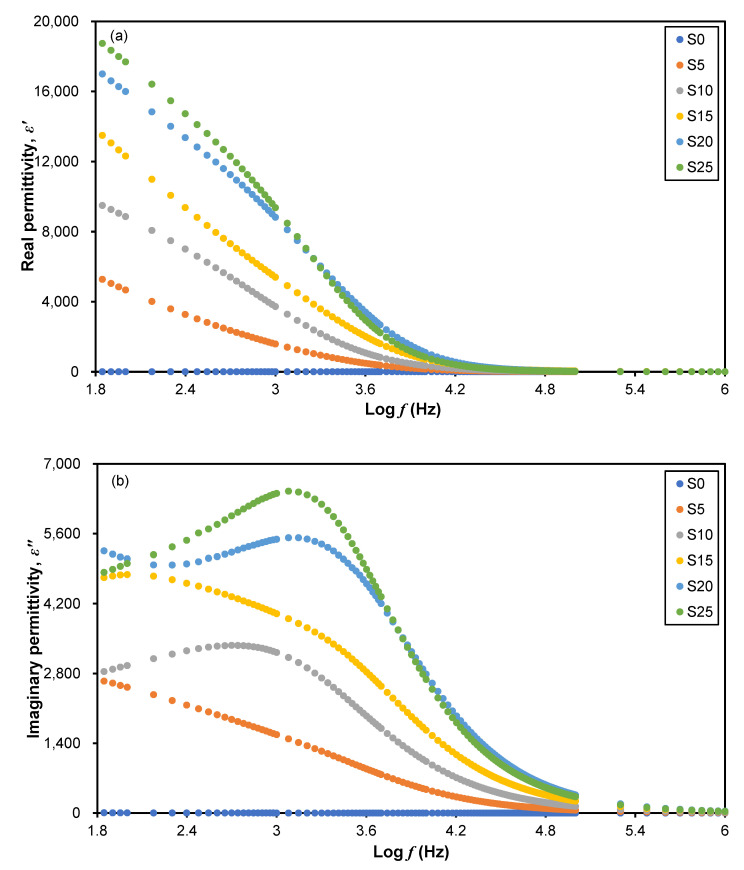
Plot of (**a**) real permittivity, *ε*′, and (**b**) imaginary permittivity, *ε*″, as a function of frequency for a 3D printed plant-based GPE system.

Impedance data obtained from the EIS measurements can be transformed into permittivity plots to evaluate the dielectric properties of 3D printed plant-based GPEs. Figure 6a and Figure 6b, respectively, show plots of real (*ε*′) and imaginary (*ε*″) permittivity as a function of frequency. The dielectric constant (*ε_r_*) for all samples can be taken at the highest frequency, in this work at 100 kHz. In Figure 6, *ε*′ and *ε*″ fluctuate with different LiClO_4_ concentrations against frequency. The values of *ε*′ and *ε*″ are high and reflect the dispersion behavior at low frequencies due to the polarization effect at the electrode/electrolyte interface [37]. In general, it can be seen that *ε_r_* decreases as the frequency increases in all samples. This decrement can be explained by the lesser ion diffusion, with insufficient energy and time for the dipole molecules of the system to align themselves in the electric field direction [38].

As shown in Figure 5, the S20 electrolyte has the highest conductivity in line with the greatest *ε*′ and *ε*″ of the sample. This is due to the accumulation of charges near the electrodes leading to the electrode polarization which causes the tendency of the dipoles in the macromolecules to align themselves in the applied field direction [39]. When the salt concentration increases to 20 wt.%, the values of *ε*′ and *ε*″ increase, which may be attributed to the increase in charge carriers. When more LiClO_4_ is added, more undissociated salt forms ions, resulting in an increase in the electrolyte’s stored charge which causes an increase in *ε_r_* values [40]. Meanwhile, a higher frequency results in a faster periodic reversal rate of the electric field which reduces *ε_r_*. There is also no additional ion diffusion in the field direction [41].

**Figure 7:**
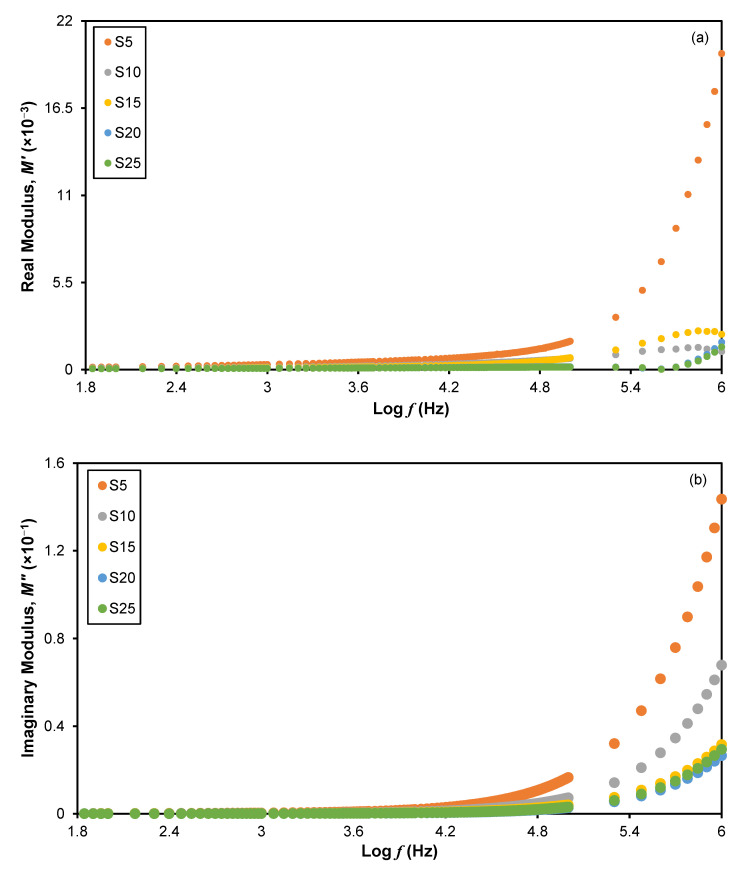
Plot of (**a**) real electrical modulus, *M*′, and (**b**) imaginary electrical modulus, *M*″, as a function of frequency for a 3D printed plant-based GPE system.

To further understand the dielectric behaviors of a 3D printed plant-based GPE system, the dielectric modulus was analyzed. The electric modulus components, *M*′ and *M*″, against log frequency of the prepared sample are shown in Figure 7. It can be seen that *M*′ and *M*″ increase dramatically when the frequency is increased, which is in accordance with the results reported previously [42]. Consequently, it was shown that the plant-based GPEs were ionic conductors, and the presence of charge carriers may influence ionic conductivity. *M*′ and *M*″ both have quantities that prone to zero with a lengthy tail when they are at low frequencies [43]. The tail finds the capacitance between the electrodes. This is because the polarization effect is minimal as there is capacitance in the electrolyte [44].

### 3.2. Fourier Transform Infrared (FTIR)

**Figure 8:**
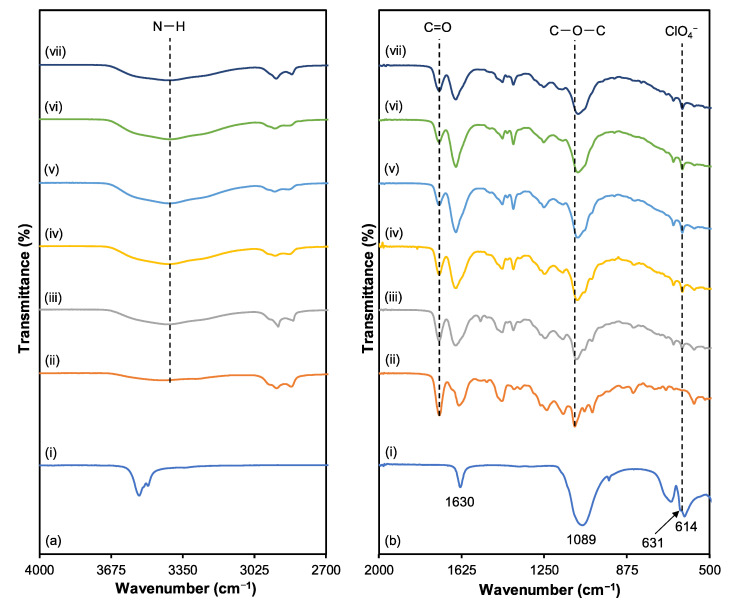
FTIR spectrum from (**a**) 4000 cm^−1^ to 2700 cm^−1^ and (**b**) 2000 cm^−1^ to 500 cm^−1^ for (i) LiClO_4_ salt, (ii) S0, (iii) S5, (iv) S10, (v) S15, (vi) S20 and (vii) S25 electrolytes.

The FTIR spectrum was investigated to evaluate the effect of salts on 3D printed plant-based GPE structures in terms of polymer electrolyte complex formation in the 500 to 4000 cm^−1^ regions. Figure 8 depicts the FTIR spectra of 3D printed plant-based GPE at various LiClO_4_ concentrations. Table 6 lists the functional groups with corresponding wavenumbers. The carbonyl (C=O) (1732–1727 cm^−1^), ether and ester (C–O–C) (1115–1093 cm^−1^), and amine (N–H) (3431–3419 cm^−1^) groups are of interest since they correlate to electronegativity atoms in the molecule of plant-based GPE. It is reported that nitrogen and oxygen atoms in the polymer matrix are accountable for interacting with Li^+^ ions from doping salt to produce polymer electrolyte complexes [45]. Complexation between Li^+^ ions with nitrogen and oxygen atoms of the corresponding molecules can be observed from changes in band wavenumber and/or intensity.

Based on Table 6, increasing the concentration of LiClO_4_ salt downshifts the peak of the C=O group of a plant-based GPE to a lower wavenumber, from 1732 cm^−1^ (S0 electrolyte) to 1726 cm^−1^ (S25 electrolyte). Furthermore, the C=O band of a plant-based GPE in the absence of Li^+^ produced an intense, strong, and sharp peak. The non-hydrogen bonded C=O exhibits a decrease in intensity as well. The intensity of the C=O band in the S25 electrolyte is lower than that of a pure plant-based GPE (S0 electrolyte). This finding implies that the interaction between carbonyl and Li^+^ salt weakens the C=O bond [46] and allows Li^+^ the sharing of electron density with the oxygen atom. The oxygen atom serves as an electron donor to Li^+^ ion to develop polymer electrolyte complexes. As the electron density of the carbonyl group decreases, the vibrational energy of the groups decreases. Therefore, the C=O band shifts to a lower wavenumber [47]. The band corresponding to the C–O–C functional group of plant-based GPE at 1115 cm^−1^ is observed to downshift as LiClO_4_ is added and is at 1093 cm^−1^ when 25 wt.% of LiClO_4_ is added. The shift of the (C–O–C) and the C=O bands can be attributed to significant intermolecular interactions between oxygen atoms and lithium ions in a plant-based GPE host polymer. It has been suggested that the oxygen atoms on the C=O and C–O–C groups in the plant-based GPE serve as electron donors to Li^+^, forming a coordination bond between the two atoms.

The non-hydrogen bonded N–H peak of pure plant-based GPE (S0 electrolyte) at 3419 cm^−1^ shifts to a higher wavenumber with increasing salt concentration. The peak becomes broader as the concentration of LiClO_4_ increases. The interaction of the N atom of the −NH group with free Li^+^ may provide an explanation for this event. Electron ion pairs and cation interactions on the nitrogen atom cause the N–H bond to weaken [48]. It may also be due to the interaction between the cations and the unpaired electrons of the carbonyl oxygen atom, which weakens the H-bond. Based on these FTIR results, it can be concluded that the interaction between the host polymer and salt occurs in the hard segment (C=O and N–H) and soft segment (C–O–C) of plant-based GPE. Following the introduction of salt into the polymer host, the overall IR spectra display a pattern similar to that seen in earlier studies [49].

Based on Figure 8, a minor peak at 623 cm^−1^ indicates the ClO_4_^−^ band once LiClO_4_ starts to dope into a 3D printed plant-based GPE. This is probably because ClO_4_^−^ is spectroscopically free [50]. Figure 8a depicts the vibrational frequencies of LiClO_4_ in the bands of 623, 630, 1089, 1368, and 1630 cm^−1^ corresponding to the internal vibrational modes of the ClO_4_^−^ anion. The ClO_4_^−^ band at 614 cm^−1^ corresponds to the spectroscopically “free” ClO_4_^−^, while the small shoulder at 631 cm^−1^ indicates the existence of contact ion pairs [51]. Rajendran et al. [52] reported that pure LiClO_4_ exhibits a vibrational peak at 1630 cm^−1^. In this work, a band of LiClO_4_ at 1630 cm^−1^ can be assigned to the characteristic of LiClO_4_. The overall results of FTIR spectroscopy show that incorporating LiClO_4_ into a 3D printed plant-based GPE leads to various interactions that can alter the microstructure of the polymer. In addition, FTIR proves that polymer and salt form a complexation through the SLA method of 3D printing.

**Figure 9:**
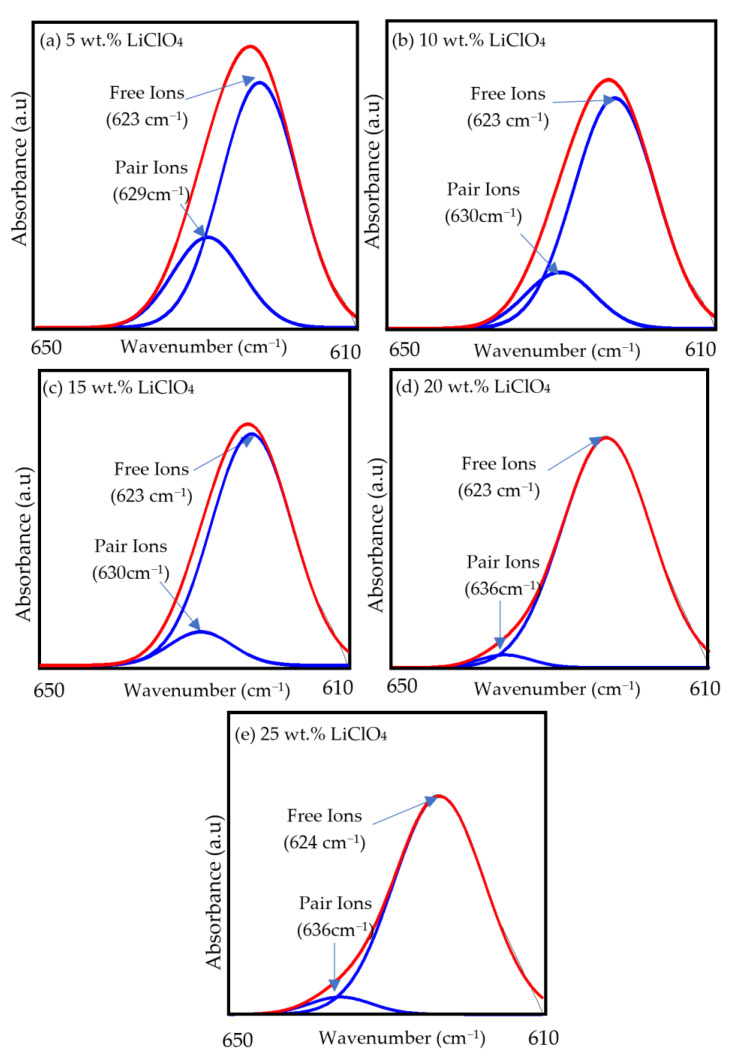
FTIR deconvolution of 3D printed plant-based GPEs with various LiClO_4_ contents between 610 and 650 wavenumbers.

Figure 9 shows the FTIR deconvolution of a band between 610 and 650 cm^−1^. The formation of free ions in a 3D printed plant-based GPE was investigated using FTIR deconvolution. Equations (6) and (7) were used to estimate the percentage of free ions and ion pairs from the deconvoluted FTIR region. In a previous study, the free ion and ion pair of ClO_4_^−^ were observed at 600 cm^−1^ and 650 cm^−1^ [53]. In this work, the peaks at 623 cm^−1^ and 624 cm^−1^ refer to free ions, while the peaks at 629 cm^−1^, 630 cm^−1^ and 636 cm^−1^ suggest ion pairs. The percentage of free ions was seen to increase from 74.84% in the S5 electrolyte to an optimum of 96.80% in the S20 electrolyte. This is due to the increase in the rate of free ions as the concentration of LiClO_4_ increases. This indicates that the increment in LiClO_4_ content generates the formation of free ions, resulting in an increase in charge carriers that could improve the electrolyte ionic conductivity [54]. Table 7 shows the percentage of free ions and ion pairs for 3D printed plant-based GPEs determined from FTIR deconvolution.

**Figure 10:**
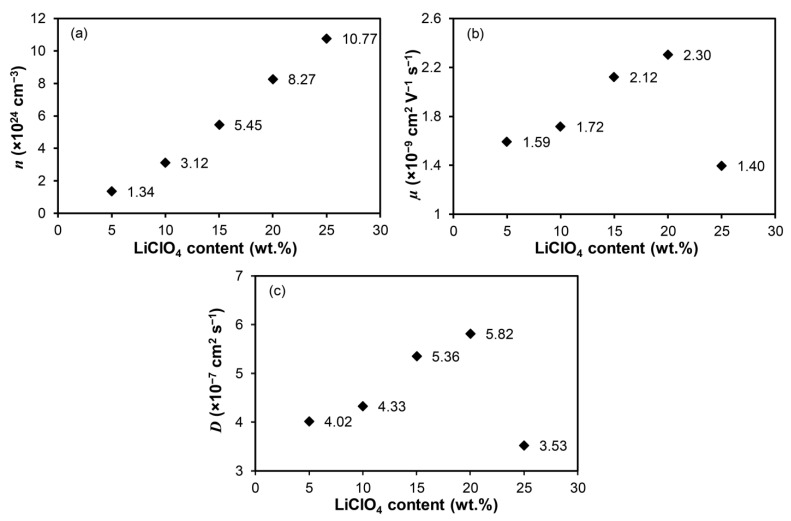
Variation of charge carrier (**a**) number density, *n*, (**b**) mobility, *μ*, and (**c**) diffusion coefficient, *D* for 3D printed plant-based GPE with various LiClO_4_ contents.

Ionic conductivity is found to be correlated with charge carrier number density (*n*), mobility (*μ*), and diffusion coefficient (*D*). Figure 10 depicts the *n*, *μ*, and *D* at different salt concentrations that influence the variation of ionic conductivity. From Figure 10a, it can be seen that *n* increases linearly from 1.34 × 10^24^ cm^−3^ to 10.8 × 10^24^ cm^−3^ as the amount of LiClO_4_ in the 3D printed plant-based GPE increases from 5 wt.% to 25 wt.%. The increase in *n* can be attributed to the increase in salt concentration that dissociates more free ions in the polymer matrix. The mobility of charge carrier is observed to increase gradually from the S5 electrolyte to the S20 electrolyte but decreases beyond the S20 electrolyte (referring to the S25 electrolyte) as illustrated in Figure 10b. The *D* in Figure 10c follows the same pattern as *μ*.

The decrease in n *μ* and *D* for samples with salt concentration beyond 20 wt.% can be attributed to the high number of charge carriers in the electrolyte. When more salt is added into the GPE, there is a significant number of free ions in the plant-based GPE complex. This leads to major collisions between ions in the electrolyte system due to the limited space results in a decrease in *µ* and *D* of charge carriers in the electrolyte. The conductivity of plant-based 3D printed GPE is dropped at higher salt concentrations. This can be attributed to the increase in ion collisions in the polymer matrix, thereby impacting the overall electrolyte conductivity since the conductivity is influenced by both charge carrier number density and mobility.

### 3.3. Transference Number Measurement (TNM)

**Figure 11:**
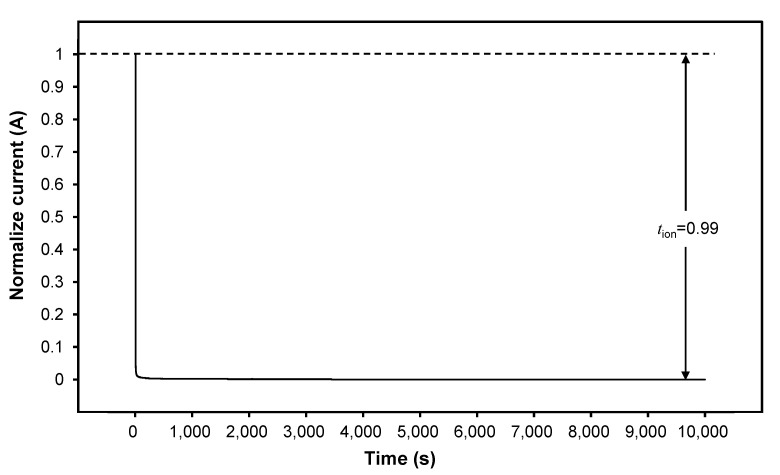
Normalized dc polarization of the S20 electrolyte.

The purpose of performing a transference number measurement (TNM) is to determine whether the conductivity of a 3D printed plant-based GPE is controlled primarily by ions or electrons. It should be emphasized that in GPE, liquid-like ion transport occurs. Thus, no electronic transport should be expected [55]. In liquids, polymers, or GPEs, the total number of ionic transports is strongly influenced by both cationic and anionic mobilization. As depicted in Figure 11, the initial current drops over time due to the depletion of ionic species in the plant-based GPE, and it then becomes constant after it is completely depleted [56]. The number of ions was calculated using Equations (11) and (12) obtained from the TNM graph in Figure 11 and offered the highest $t_{ions}$ value of 0.99897 and the lowest $t_{ele}$ value of 0.00103 for S20 electrolyte. This indicates that the majority of charge transport in this polymer electrolyte film is due to ions, while the contribution of electrons is negligible. The role of ionic mobility in total conductivity is very important because ions, which are considered charge carriers, play an important role in device applications. A high $t_{ions}$ at lower dc voltage indicates stable ionic conduction and is an excellent indicator of device efficiency [57]. In addition, the ionic mobility of cations that serve as ion conductors impacts the conductivity of the 3D printed plant-based GPE. Thus, it shows that ions play a major role in charge transfer in this polymer electrolyte film, while electrons play a minor role.

### 3.4. Thermal Gravimetric Analysis (TGA)

**Figure 12:**
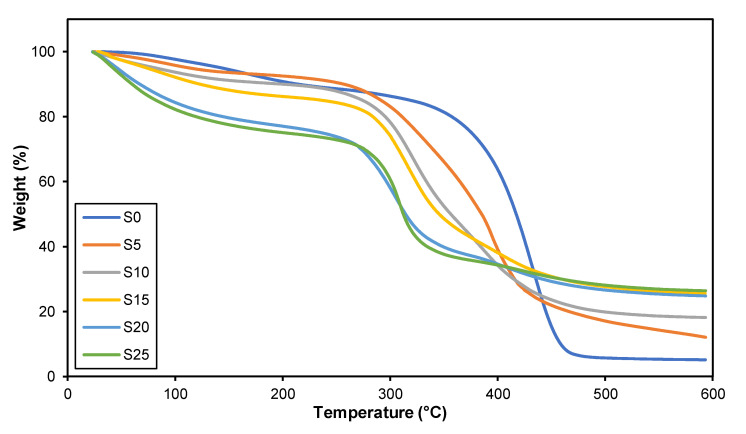
TGA analysis of 3D printed plant-based GPE with various LiClO_4_ contents.

The TGA thermograms of the 3D printed plant-based GPE with various LiClO_4_ salt concentrations are presented in Figure 12. It shows the change in sample weight with increasing temperature. In this investigation, the decomposition of plant-based GPE with LiClO_4_ is divided into two stages as shown in Table 8. The breakdown of LiClO_4_ salt is at the first stage ($T_{d\max1}$), which occurs at temperatures between 270 and 330 °C [58]. The decomposition occurs in the first stage due to the solvent evaporation confined in the polymer electrolyte, and water exists because of the hygroscopic behavior of LiClO_4_ [59]. The solvent used is dimethylformamide (DMF), and its boiling point is 153 °C. In addition, the degradation of the polymer matrix between 330 and 460 °C is the second stage ($T_{d\max2}$). The interaction between oxygen atoms and Li^+^ ions in plant-based GPE weakens the C=O bond. This finding is related to a previous study [60]. In addition, increasing the amount of lithium salt present in a 3D printed plant-based GPE reduces the percentage of mass loss upon heating. This can be seen in Table 8 where the pure plant-based polymer (S0 electrolyte) residue is 5.145% while the 5 wt.% to 25 wt.% LiClO_4_ plant-based GPE remains as residue in the range of 12.076% to 26.398% of its weight. It can be concluded that the more LiClO_4_ in the plant-based GPE, the higher the temperature required to break the polymer chains in the plant-based GPE.

### 3.5. Differential Scanning Calorimetry (DSC)

**Figure 13:**
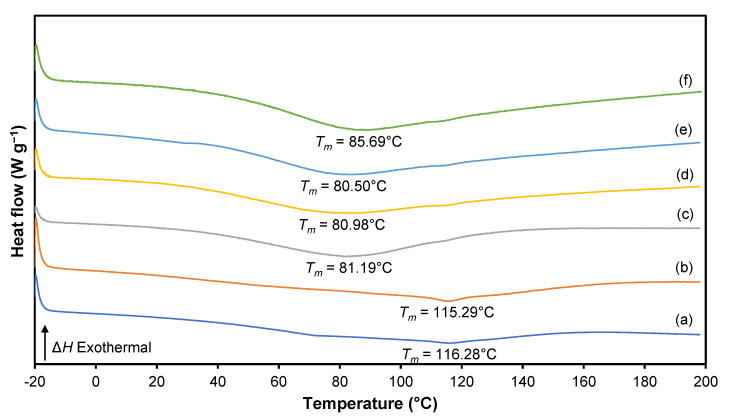
DSC thermogram for (**a**) S0, (**b**) S5, (**c**) S10, (**d**) S15, (**e**) S20 and (**f**) S25 electrolytes.

**Figure 14:**
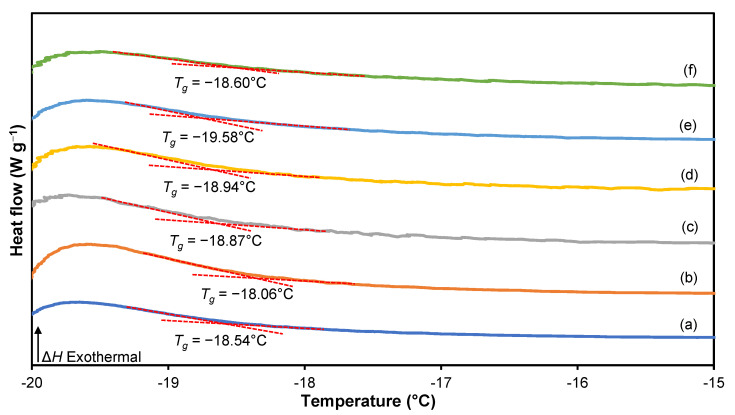
Position of glass transition temperature, *T_g_* for (**a**) S0, (**b**) S5, (**c**) S10, (**d**) S15, (**e**) S20 and (**f**) S25 electrolytes.

The glass transition temperature (*T_g_*) and endothermic melting peaks (*T_m_*) of 3D printed plant-based GPEs were analyzed using differential scanning calorimetry (DSC) and are presented in Figure 13 and Figure 14, respectively. The *T_g_* of the salt-free 3D printed plant-based GPE is at −18.54 °C, which is similar to that previously reported [61]. An increase in the ionic conductivity of the polymer electrolyte is associated with a decrease in *T_g_* and *T_m_* of the polymer host. The plasticizing effect of Li salt in the polymer matrix causes a lower *T_g_* value that can be observed after a 20 wt.% LiClO_4_ is used.

**Figure 15:**
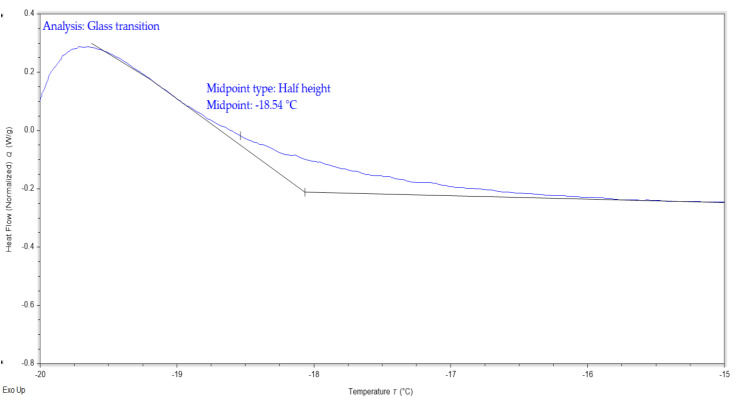
TRIOS software 5.1.1 is used to perform DSC analysis on pure 3D-printed plant-based GPE.

Referring to Table 9, the value of *T_m_* decreases with an increasing LiClO_4_ concentration from 5 to 20 wt.%. The addition of salt to the 3D printed plant-based GPE reduces the dipole–dipole interactions between the polymer chains, allowing ions easier moving throughout the polymeric chain network in response to an applied electric field. Interestingly, it is found that GPE with a 20 wt.% LiClO_4_ causes a decrease in *T_m_*, indicating that the increase in salt content hinders the dipole–dipole interaction. The addition of Li salt further constrains the plant-based GPE chain and reduces the interchain bond connection. TRIOS software 5.1.1 was used to perform DSC analysis on pure 3D-printed plant-based GPE as depicted in Figure 15.

### 3.6. Field Emission Scanning Electron Microscope (FESEM)

**Figure 16:**
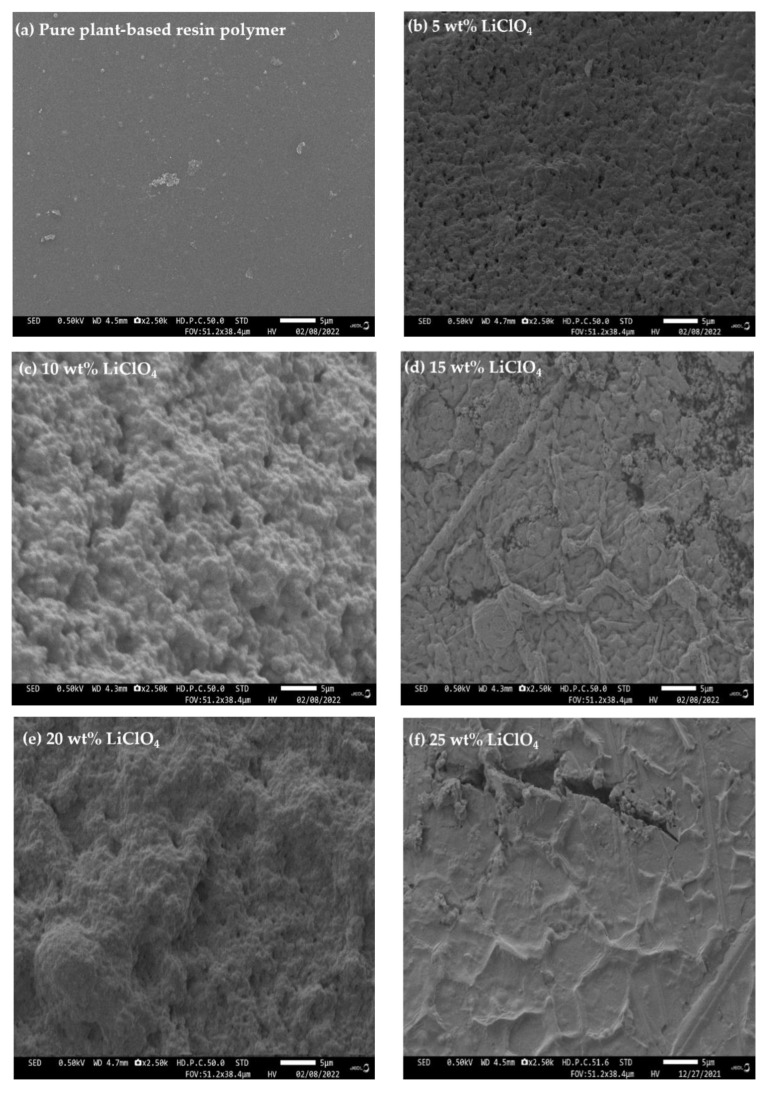
FESEM micrograph of plant-based GPE with various LiClO_4_ concentrations.

Figure 16 depicts the FESEM micrographs of the cross-sectional area of plant-based GPE with various LiClO_4_ salts concentrations. The cross-section surface of a pure plant-based polymer (S0 electrolyte) in Figure 16a is relatively smooth, while the cross-sections of a plant-based GPE with 5 to 25 wt.% LiClO_4_ salt in Figure 16b–f depicts a porous image. The formation of micropores occurs when salt is applied to the polymer complex system. This effect occurs due to the complex interactions of the solvent, lithium salt and polymer. Micropores in the polymer–salt systems increase the mobility of ions by providing and developing multiple pathways for ion transport. However, Figure 16f shows that the addition of a 25 wt.% LiClO_4_ salt can create agglomeration, which hinders the movement of Li-ion in the plant-based GPE, resulting in reduced ionic conductivity. The mobility (*μ*) of charge carriers decreases beyond 20 wt.% LiClO_4_ from 2.30 × 10^−9^ cm^2^ V^−1^ s^−1^ to 1.40 × 10^−9^ cm^2^ V^−1^ s ^−1^ as shown in Figure 10b.

### 3.7. Pure Plant-Based Polymer Biodegradation Test

**Figure 17:**
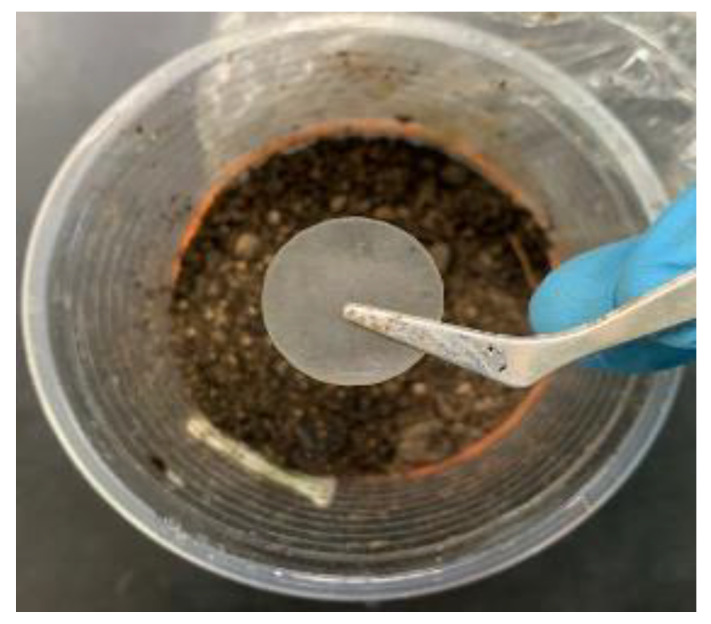
Appearance of pure 3D printed plant-based film before the soil burial test.

**Figure 18:**
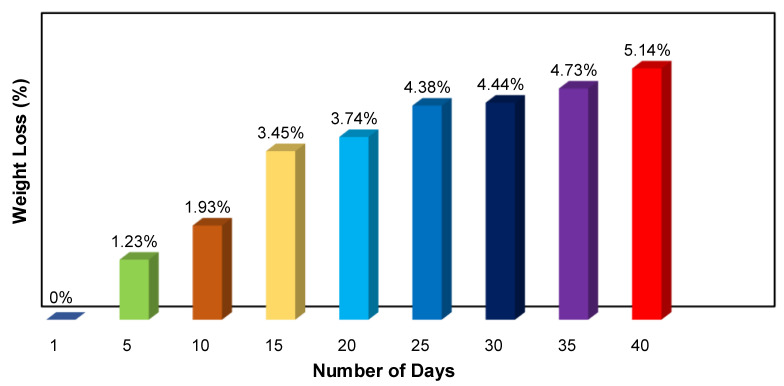
Weight loss of pure 3D printed plant-based film after 40 days.

As an environmental issue, pure 3D printed plant-based film is subjected to soil bio-degradability tests to determine its structure and the rate of its decomposition. Figure 17 shows the visual appearance of pure 3D printed plant-based film before the soil burial test. The peat moss soil used is a dark brown fibrous byproduct of sphagnum moss and all other organic components that have decayed over thousands of years in peat bogs. Moreover, the pH of the soil is 7.62. The nitrogen (N), phosphorus (P) and potassium (K) content of the soil is approximately 83.31 kg ha^−1^, 58.3 kg ha^−1^ and 75 kg ha^−1^, respectively. Figure 18 shows a bar graph indicating the percentage of weight loss of a pure 3D printed plant-based film over time, and the film shows a significant decay of 1.23% during the starting point, then decays continuously reaching a degradation order of around 5.14% by the end of the 40th day. The rate and degree of biodegradation of the polymer film are mainly determined by the soil conditions and the chemical composition of the polymer. The polymer film loses weight due to the biodegradation process when it is buried in alkaline-rich soil. Soil microorganisms gradually migrate, cover and decompose the entire surface of the 3D printed plant-based film [62]. Table 10 shows the weight loss of pure plant-based polymer over 40 days of the soil burial test.

## 4. Conclusions

The 3D printed plant-based GPE was fabricated by the stereolithography (SLA) photopolymerization technique with various concentrations of lithium perchlorate (LiClO_4_). For sample analysis, 3D printed plant-based GPE productions exhibited optimal conductivity at a 20 wt.% LiClO_4_ concentration due to the increase in concentration of charge carriers. FTIR showed that the interaction of Li salts with the polymer matrix shifted the functional groups of C=O and C–O–C to a lower wavenumber, while the N–H functional group shifted to a higher wavenumber. The N–H band in the region between 3800 and 3100 cm^−1^ was observed to broaden when the LiClO_4_ content increased from 0 wt.% to 25 wt.% in plant-based GPE. The deconvolution of the FTIR spectrum represented an increase in free ion percentage with 96.80% free ions obtained in the sample with a 20 wt.% LiClO_4_ (S20 electrolyte). The highest ionic transference number obtained was 0.9989 at the S20 electrolyte. TGA studies revealed that increasing the amount of LiClO_4_ in plant-based GPE required higher temperatures to break the polymer chains in the plant-based GPE films in contrast to pure plant-based polymer. In addition, DSC identified the glass transition temperature (*T_g_*) and endothermic melting peaks (*T_m_*) of samples. The glass transition temperature (*T_g_*) of −18.54 °C was obtained for undoped 3D printed plant-based GPE. Moreover, the creation of micropores was confirmed by FESEM when salt was applied to the complex system. Ion mobility was increased by the micropores in the polymer–salt matrix because they provide and create more pathways for ion transport. A biodegradation test was performed, which revealed that the pure plant-based polymer lost between 0% and 5.14% of its weight due to microorganisms, starting from 0.1711 g to 0.1623 g in 40 days when exposed to the environment. In conclusion, the LiClO_4_ salt confined in 3D printed plant-based GPE provided more advantages and benefits in battery applications. The SLA technique also demonstrated superior accuracy and shape flexibility for the 3D printed plant-based GPE fabrication compared to other conventional approaches.

## Figures and Tables

**Table 1:** Ingredients and chemical composition of Anycubic plant-based resin [29].

Ingredients	Compositions (%)
Concentration of fatty acids, soya, epoxidized, Bu esters	45
Isooctyl acrylate (C_11_H_20_O_2_)	30
2-((2,2-Bis(((1-oxoallyl)oxy)methyl)butoxy)methyl)-2-ethyl-1,3-propanediyl diacrylate	15
2-hydroxy-1-(4-(4-(2-hydroxy-2-methylpropionyl)benzyl)phenyl)-2-methylpropan-1-one	5
Polychloro copper phthalocyanine	5

**Table 2:** Regular resin versus plant-based resin.

Major Characteristics	Regular Resin	Plant-Based Resin
Resin odor	Smelly	Slightly
Ingredient	Industrial chemical	Soy oil
Washing odor	Pungent	Detergent smell
Range of wavelength	405 nm	355–410 nm
Eco-friendly	Difficult to degrade	Biodegradable

**Table 3:** Designation and sample composition of plant-based resin GPE system.

Designation	LiClO_4_ Content (wt.%)	Plant-Based Resin (g)	DMF (g)	LiClO_4_ (g)
S0	0	2.00	2.00	0
S5	5	2.00	2.00	0.21
S10	10	2.00	2.00	0.44
S15	15	2.00	2.00	0.71
S20	20	2.00	2.00	1.00
S25	25	2.00	2.00	1.33

**Table 4:** Anycubic Photon S settings with corresponding parameters.

Anycubic Photon S Settings	Parameters
Layer height	0.5 mm
Exposure time	10 s
Off-time	6.5 s
Exposure on 8 bottom layers	70 s
Distance of Z-lift	6 mm
Speed	1 mm s^−1^

**Table 5:** Bulk resistance and ionic conductivity of 3D printed plant-based GPEs.

Sample	*R_b_* (Ω)	Ionic Conductivity, *σ* (S cm^−1^)
S0	7.82 × 10^5^	2.27 × 10^−8^
S5	5.18 × 10^1^	3.42 × 10^−4^
S10	2.09 × 10^1^	8.56 × 10^−4^
S15	9.07	1.85 × 10^−3^
S20	8.86	3.05 × 10^−3^
S25	9.95	2.41 × 10^−3^

**Table 6:** Functional groups with a corresponding wavenumber for 3D printed plant-based GPEs.

Functional Groups	Wavenumber (cm^−1^)						
	LiClO_4_	S0	S5	S10	S15	S20	S25
C=O	-	1732	1731	1730	1729	1727	1727
N−H	-	3419	3421	3423	3426	3429	3431
C−O−C	-	1115	1110	1106	1101	1098	1093
LiClO_4_ characteristics	1630	-	1630	1630	1630	1630	1630
ClO_4_ asymmetric vibration	1089	-	1089	1089	1089	1089	1089
LiClO_4_ ion pairs	631	-	629	630	630	636	636
ClO_4_^−^ free ions	614	-	623	623	623	623	624

**Table 7:** Percentage of free ions and ion pairs for 3D printed plant-based GPEs determined from FTIR deconvolution.

Sample	Percentage of Free Ions (%)	Percentage of Ion Pairs (%)
S5	74.84	25.16
S10	82.86	17.14
S15	89.85	10.15
S20	96.80	3.20
S25	94.75	5.25

**Table 8:** TGA analysis of a 3D-printed plant-based GPE system.

Sample	*T*_*d*max1_ (°C) (First Stage)	*T*_*d*max2_ (°C) (Second Stage)	Weight Change (%)	Residue (%)
S0	-	457.00	94.855	5.1450
S5	337.00	423.00	87.924	12.076
S10	286.99	397.63	81.846	18.154
S15	285.17	373.62	74.391	25.609
S20	273.24	350.31	75.194	24.806
S25	289.45	329.78	73.602	26.398

**Table 9:** Values of *T_g_* and *T_m_* for a 3D printed plant-based GPE system.

Sample	*T_g_* (°C)	*T_m_* (°C)
S0	−18.54	116.28
S5	−18.06	115.29
S10	−18.87	81.19
S15	−18.94	80.98
S20	−19.58	80.50
S25	−18.60	85.69

**Table 10:** Weight of pure 3D printed plant-based film and its percentage weight loss after being buried from Day 1 to Day 40.

Days	Weight of Pure Plant-Based Polymer (g)	Weight Loss of Pure Plant-Based Polymer after Burial (%)
1	0.1711	-
5	0.1690	1.23
10	0.1678	1.93
15	0.1652	3.45
20	0.1647	3.74
25	0.1636	4.38
30	0.1635	4.44
35	0.1630	4.73
40	0.1623	5.14

## Data Availability

Data are contained within the article.
